# Detection of murine post-pneumonectomy lung regeneration by ^18^FDG PET imaging

**DOI:** 10.1186/2191-219X-2-48

**Published:** 2012-09-21

**Authors:** Barry C Gibney, Mi-Ae Park, Kenji Chamoto, Alexandra Ysasi, Moritz A Konerding, Akira Tsuda, Steven J Mentzer

**Affiliations:** 1 Laboratory of Adaptive and Regenerative Biology, Brigham & Women's Hospital, Harvard Medical School, Boston, MA 02115, USA; 2 Department of Radiology, Brigham & Women's Hospital, Harvard Medical School, Boston, MA 02115, USA; 3 Institute of Functional and Clinical Anatomy, University Medical Center of Johannes Gutenberg-University, Mainz 55131, Germany; 4 Molecular and Integrative Physiological Sciences, Harvard School of Public Health, Boston, MA 02115, USA; 5 Brigham & Women's Hospital, 75 Francis Street, Room 259, Boston, MA 02115, USA

**Keywords:** Lung, Regeneration, Computerized tomography, Positron-emission tomography

## Abstract

**Background:**

An intriguing biologic process in most adult mammals is post-pneumonectomy lung regeneration, that is, the removal of one lung (pneumonectomy) results in the rapid compensatory growth of the remaining lung. The spatial dependence and metabolic activity of the rodent lung during compensatory lung regeneration is largely unknown.

**Methods:**

To determine if murine lung regeneration could be detected *in vivo*, we studied inbred mice 3, 7, 14, and 21 days after left pneumonectomy. The remaining lung was imaged using microCT as well as the glucose tracer 2-deoxy-2-[^18^ F]fluoro-d-glucose (^18^FDG) and positron-emission tomography (PET). Because of the compliance of the murine chest wall, reproducible imaging required orotracheal intubation and pressure-controlled ventilation during scanning.

**Results:**

After left pneumonectomy, the right lung progressively enlarged over the first 3 weeks. The cardiac lobe demonstrated the greatest percentage increase in size. Dry weights of the individual lobes largely mirrored the increase in lung volume. PET/CT imaging was used to identify enhanced metabolic activity within the individual lobes. In the cardiac lobe, ^18^FDG uptake was significantly increased in the day 14 cardiac lobe relative to preoperative values (*p* < .05). In contrast, the ^18^FDG uptake in the other three lobes was not statistically significant at any time point.

**Conclusions:**

We conclude that the cardiac lobe is the dominant contributor to compensatory growth after murine pneumonectomy. Further, PET/CT scanning can detect both the volumetric increase and the metabolic changes associated with the regenerative growth in the murine cardiac lobe.

## Background

The capacity for adult tissue regeneration has heightened interest in the longitudinal and minimally invasive visualization of these complex biologic processes [1]. Molecular imaging of dynamic processes such as tissue regeneration provides an opportunity not only to characterize the biologic process at the cellular and molecular level but also to measure the impact of therapeutic interventions [2-4].

The dominant clinical and experimental approach to characterizing biologic processes has utilized the glucose analogue tracer 2-deoxy-2-[^18^ F]fluoro-d-glucose (^18^FDG) and positron-emission tomography (PET). FDG-PET can discriminate between tissues with normal and increased glucose metabolism. Recently, imaging systems that combine PET and computerized tomography (CT) have permitted the spatial integration of both metabolic and anatomic information.

FDG-PET has been used to characterize a variety of nonmalignant processes. Cardiac FDG-PET imaging, using electrocardiogram gating, has been shown to identify areas of myocardial infarction as well as define functional parameters such as ejection fraction [5]. The activity of gastrointestinal inflammatory diseases, such as graft-versus-host disease, has been effectively monitored using FDG-PET [6]. Similarly, the increased glucose metabolism associated with liver regeneration has been demonstrated by FDG-PET after radiofrequency ablation [7] and partial hepatectomy [8]. Despite the experimental advantage of low baseline glucose metabolism and the desirability of longitudinal noninvasive monitoring, FDG-PET has not been routinely applied to the study of nonmalignant processes in the lung.

A particularly intriguing biologic process in the lung is post-pneumonectomy lung regeneration. In most adult mammals, the removal of one lung (pneumonectomy) results in the rapid compensatory growth of the remaining lung [9]. Pneumonectomy in mice results in the growth of the remaining lung to near baseline levels (two lungs) within 3 weeks [10]. Recent microCT data indicate differential lobar growth and angiogenesis in the regenerating lung [11]. In this study, we used FDG-PET and microCT scanning to investigate the spatial dependency of enhanced metabolic activity during post-pneumonectomy compensatory lung growth.

## Methods

### Mice

C57/B6 mice (Jackson Laboratory, Bar Harbor, ME, USA), 22 to 30 g, were used in all experiments. The care of the animals was consistent with the guidelines of the American Association for Accreditation of Laboratory Animal Care (Bethesda, MD, USA).

### Orotracheal intubation and anesthesia

The animal was suspended by the incisors, and the tongue gently retracted anteriorly. A 20 G angiocatheter (BD Insyte, Sandy, UT, USA) was passed between the vocal cords under direct illumination. For surgical procedures, anesthesia was provided with a mixture of 100 mg/kg ketamine (Fort Dodge Animal Health, IA, USA) and 0.6 mg/kg xylazine (Phoenix Scientific, Inc., St. Joseph, MO, USA) administered intraperitoneally (IP) prior to intubation [12]. For imaging studies, the mouse was pre-oxygenated for 5 min with 100% FiO_2_ and induced with 5% isoflurane at 2 L/min prior to intubation [13]. The animal was then maintained on 1.5% isoflurane at 2 L/min during imaging.

### Pneumonectomy

Anesthesia for pneumonectomy was a mixture of 100 mg/kg ketamine (Fort Dodge Animal Health) and 0.6 mg/kg xylazine (Phoenix Scientific, Inc., St. Joseph, MO, USA) administered IP. The mice were intubated with a 20 G angiocatheter (BD Insyte, Sandy, UT, USA) and ventilated on a FlexiVent ventilator (SCIREQ, Montreal, Quebec, Canada) at standard settings of 200 bpm, 10 ml/kg, a pressure limit of 30 cmH_2_O, and a PEEP of 3 cmH_2_O. A left thoracotomy was performed through the fifth intercostal space, the hilum was ligated with a 5-0 silk tie (Ethicon, Somerville, NJ, USA), and the lung was sharply excised distal to the ligature. A recruitment maneuver was performed to recruit the contralateral lung and medialize the mediastinum as the thoracotomy was closed. The mouse was extubated and transferred to a warmed cage until recovered from the anesthesia.

### CT and PET/CT scanners

CT scans alone were obtained with a GE eXplore 120 CT scanner (GE Healthcare, Fairview, CT, USA). PET/CT imaging was performed with a GE eXplore VISTA PET/CT scanner. In the PET/CT scanner, the imaging field of view was 6 cm (transverse) × 4.6 cm (axial). The system utilized two crystals, LYSO and GSO, with a different scintillation decay time. The 1.5-mm wide scintillation crystals generated high-resolution images: 1.6 mm at the center of the field of view and yields with approximately 4% count sensitivity. The data were typically acquired in list mode and whole body modes in 3D.

### PET/CT scanning protocol

The PET/CT images were obtained preoperation and on days 3, 7, 14, and 21 postoperation. After initial kinetic data were obtained, subsequent scans were obtained for 20 min after a 30-min FDG uptake period. All experiments were performed using isoflurane general anesthesia. The average injected FDG was 168 ± 41 uCi (6.2 MBq). PET images were corrected for random and scatter coincidence events and reconstructed with the ordered-subsets expectation-maximization algorithm using 16 subsets and two iterations. No attenuation correction was applied. The average weight of the mice was 26.1 ± 1.6 g. Fasting times were 2 h. Standardized uptake values (SUV) were calculated at 30 min post-injection according to the formula [14]:

$$\text{SUV}=\frac{c}{\left(A/W\right)}$$

where *c* was the activity concentration in a lung region of interest measured by the PET scanner (Bq/ml), *A* was the injected activity (Bq), and *W* was the mouse body weight in grams.

### Lung weights

After euthanasia, the remaining right lung was removed, blotted, and weighed [11]. The weight of the individual lobes was measured and expressed as a ratio of lung weight to animal weight. The lung weight index (LWI), in grams per kilogram body weight, was calculated to correct for variability in animal size.

### Statistical analysis

The statistical analysis was based on measurements in at least three different mice. The unpaired Student's *t* test for samples of unequal variances was used to calculate statistical significance. The data were expressed as mean ± one standard deviation. The significance level for the sample distribution was defined as *p* < .05.

## Results

### Mouse PET/CT chest imaging

**Figure 1:**
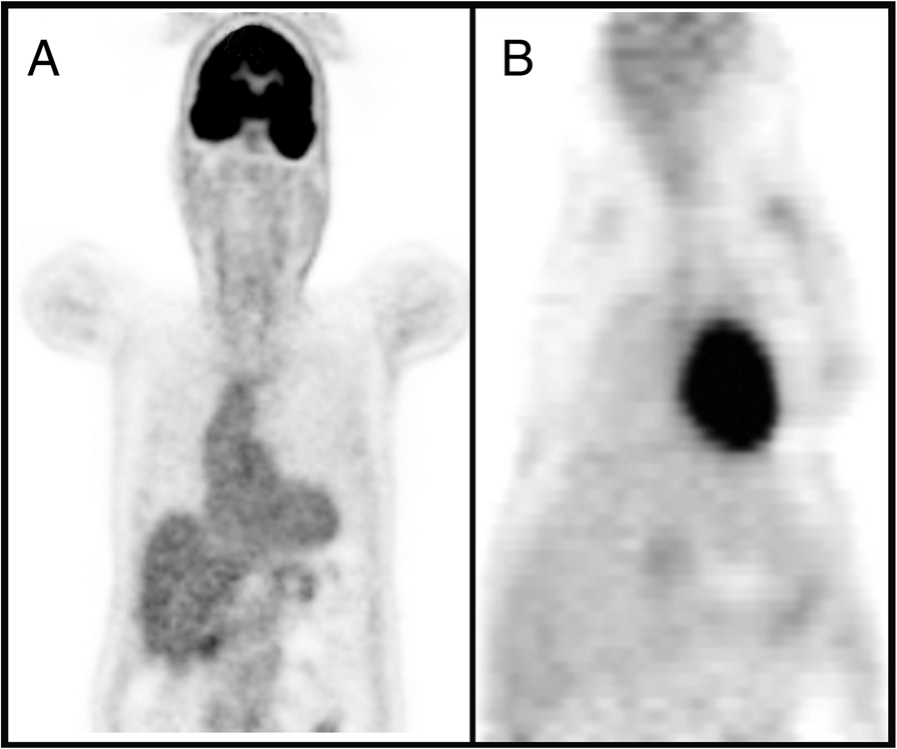
**Coronal FDG-PET imaging comparison of a normal human (A) and mouse (B). **Note the similar cardiac uptake, but diminished mouse brain uptake potentially related to the use general anesthesia.

**Figure 2:**
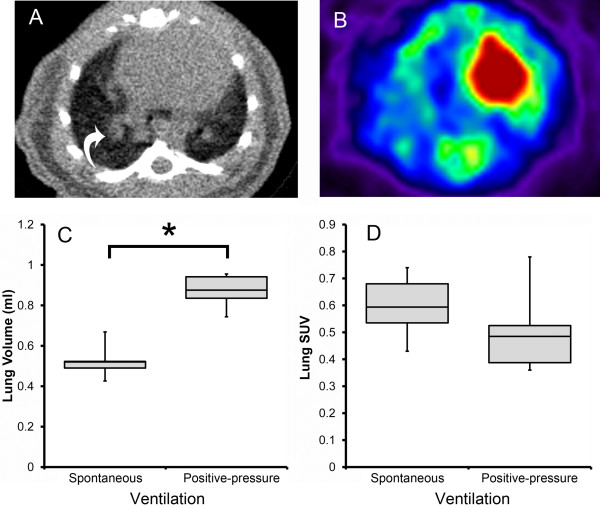
**Comparison of CT (A) and FDG-PET (B) images of the sedated mouse without positive-pressure ventilation. **The transverse images were obtained with isoflurane anesthesia using a nose cone (‘spontaneous’ ventilation) (**A, B**). The absence of ventilation resulted in pulmonary congestion (**A**, arrow) and presumed increase in PET image noise and errors in tracer uptake (**B**). (**C**) Comparison of CT scans obtained with spontaneous ventilation and after orotracheal intubation and positive-pressure ventilation (‘positive-pressure’ ventilation) demonstrated a significant difference in lung volumes (*t* test, *p* <. 001, asterisk) (*N* = 3). (**D**) Comparison of FDG-PET scans obtained with spontaneous ventilation and positive-pressure ventilation (‘positive-pressure’ ventilation) demonstrated a trend toward lower baseline SUV levels with positive-pressure ventilation.

Despite imaging similarities between mouse and human PET/CT scans (Figure 1), the experimental differences were notable: (1) mice were fasted for less than 2 h (not 4 h), (2) the total radionuclide activity approximated 7.4 MBq (200 uCi), and (3) the mouse required general anesthesia to remain motionless. A significant feature of murine physiology is that even mild sedation results in a loss of lung volume and subsequent hypoxemia [12]. The effect of sedation on lung volumes was apparent with CT and PET imaging of the chest. CT scans of sedated mice were notable for vascular congestion and indistinct lobar anatomy (Figure 2A, B). In contrast, orotracheal intubation and positive-pressure ventilation provided reproducible CT and PET images (Figure 2C, D).

### Imaging protocol

**Figure 3:**
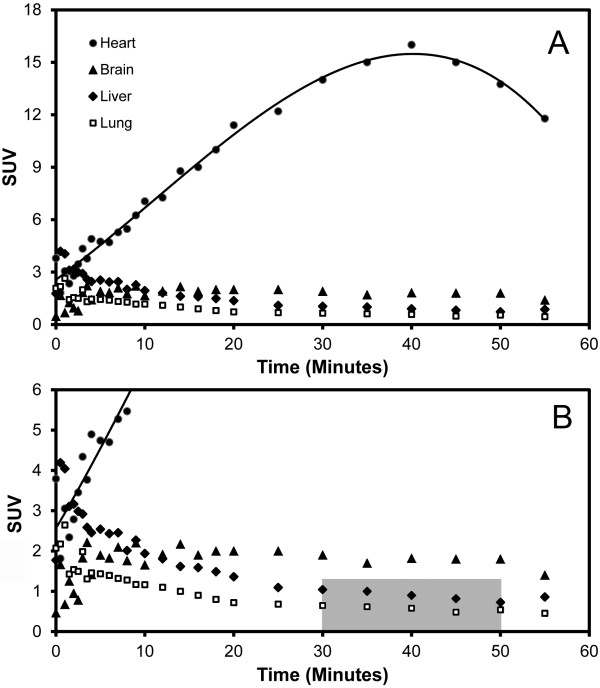
**Kinetics of tracer uptake after intravenous (tail vein) injection of FDG. **(**A**) Time-activity curve of the lung, heart, brain, and liver uptakes in a normal mouse; polynomial curve fitting (third degree) is shown for presentation purposes. (**B**) Based on the kinetic studies, most experiments used a 20-min scan after a 30-min uptake period (gray box).

To determine the optimal imaging interval, the kinetics of FDG delivery, binding, and washout were determined for the heart, brain, liver, and lung. Although there was an initial ‘first-pass’ uptake of the tracer in the lung, the FDG rapidly equilibrated to a plateau level (Figure 3A). Based on preliminary experiments, subsequent imaging protocols used a 20-min scan after a 30-min uptake period (Figure 3B, gray box). Because preliminary experiments demonstrated variable cardiac FDG uptake with ketamine (not shown), all subsequent PET scans were performed with isoflurane (1.5%) and a standard positive-pressure ventilation protocol.

### CT imaging after pneumonectomy

**Figure 4:**
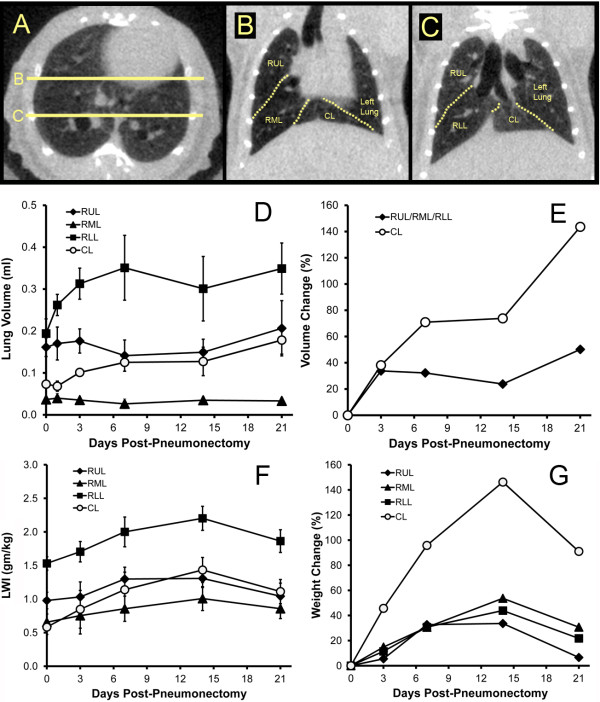
**Mouse chest CT imaging post-pneumonectomy. **Chest CT imaging in the transverse (**A**) and coronal planes (**B**, **C**) demonstrating the lobes of the right lung in a normal mouse (**A, B, C**). Based on chest CT imaging, the lobar volumes were calculated as their absolute volume (**D**) and percent volume (**E**) change in the 21 days after pneumonectomy. In comparison, the absolute weight (**F**) and percent weight (**G**) change in the four lobes were measured. CT lobar volume was calculated from representative scans of weight-matched mice. Lobar weights were measured in *N* = 3 to 5 mice at each time point. RUL, right upper lobe; RML, right middle lobe; RLL, right lower lobe; CL, cardiac lobe.

In most mice, high-resolution CT scans enabled the identification of the individual lobes of the right lung (Figure 4A, B, C). After pneumonectomy, the right lung progressively enlarged over the first 3 weeks. Of note, the cardiac lobe demonstrated the greatest percentage increase in size (Figure 4E). Because the enlarged right lung might reflect simple alveolar stretch and not true lung growth, the dry weight of the individual lobes was determined. Both lobar volume and weight increased during the 2 weeks post-pneumonectomy; the greatest percentage increase in weight occurred in the cardiac lobe (Figure 4G). Of note, there was a decrease in the LWI on day 21, reflecting a change in total body weight.

### PET imaging of the cardiac lobe

**Figure 5:**
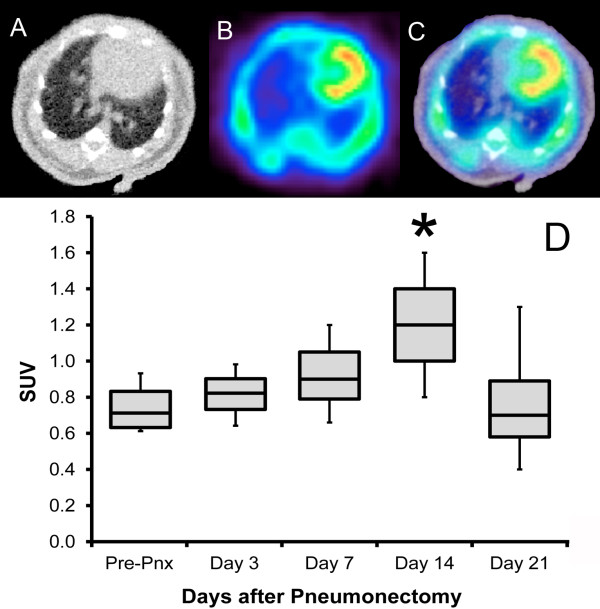
**Fusion PET/CT images (A, B, C) used to assess FDG activity in individual lobes after pneumonectomy.** The SUV (**D**) of the cardiac lobe on day 14 after pneumonectomy demonstrated significantly increased activity relative to pre-pneumonectomy controls (*p* < .05, asterisk). No other time point demonstrated significantly increased activity. *N* = 3 to 5 mice at each time point.

**Figure 6:**
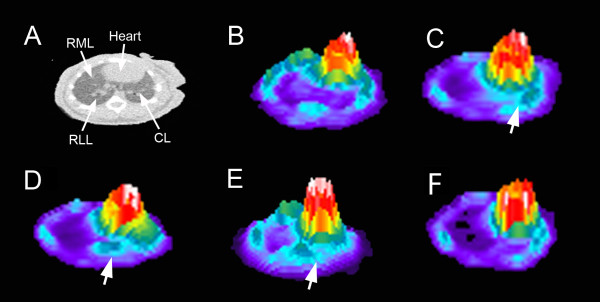
**Intensity plots of FDG uptake in the cardiac lobe at various times after pneumonectomy. **(**A**) A CT image of the transverse plane of the post-pneumonectomy lung identifying the location of the heart, right middle lobe (RML), right lower lobe (RLL), and cardiac lobe (CL). FDG-PET intensity profiles of a similar transverse plane on the day of surgery (**B**) and days 3 (**C**), 7 (**D**), 14 (**E**), and 21 (**F**) after pneumonectomy. Apparently increased uptake in the cardiac lobe is denoted (large arrow). Representative images at each time point are shown.

The increase in size and weight of the cardiac lobe suggested that FDG uptake in the cardiac lobe might be detectable by PET imaging. Using microCT scanning to identify lobar anatomy, FDG activity in each lobe was evaluated. FDG uptake was significantly increased in the day-14 cardiac lobe relative to preoperative values (*p* <. 05) (Figure 5D). In contrast, the change in FDG uptake in the other three lobes was not statistically significant at any time point. Consistent with the FDG uptake reflecting lobar growth, the time course of the FDG activity mirrored the increase in lobar weight. Intensity plots of the FDG activity in the transverse plane, reflecting the greatest cross-sectional area of the cardiac lobe, demonstrated increased activity in the cardiac lobe at 14 days after pneumonectomy (Figure 6). No significant longitudinal change in FDG uptake was demonstrated in contiguous organs such as the heart and liver (not shown).

## Discussion

In this report, we used FDG-PET and microCT imaging to quantitatively characterize compensatory lung growth after murine pneumonectomy. CT scanning demonstrated enlargement of all four lobes of the right lung, but particular growth in the cardiac lobe. The growth of the cardiac lobe was associated with significantly elevated FDG avidity; the growth in the other three lobes of the lung was not significantly different from baseline scans. We conclude that FDG-PET and microCT scanning can detect both the volumetric increase and the associated metabolic changes during compensatory growth of the cardiac lobe.

The significance of this study is that it illustrates the relevance of longitudinal spatial information in the investigation of complex biologic processes such as tissue regeneration. In the case of lung regeneration, CT scanning demonstrated differential growth in the cardiac lobe; coincident metabolic imaging with increased FDG uptake in the same region indicated that the increase in volume was not simple alveolar stretch but true lung growth. Furthermore, serial imaging defined the time course for the increased metabolic activity; notably, the peak of metabolic activity occurred 14 days after pneumonectomy. Although we recognize that post-pneumonectomy lung growth is a particularly robust example of tissue regeneration, the active proliferation of approximately 12% of all lung cells [10,11], it does highlight the utility of *in vivo* labeling of regenerating tissues. We anticipate that the potential applications will broaden as scanner resolution improves.

An experimental advantage in our study was that the PET/CT imaging was largely noninvasive, that is, it provided an opportunity to investigate regenerative processes with serial measurements and without tissue biopsies or organ harvest. Many recent reports have used the spatial resolution and sensitivity of PET/CT for stem cell tracking. These reports have generally used direct *ex vivo* labeling of stem cells with FDG [15,16] or indirect labeling using a reporter gene such as herpes simplex virus type I thymidine kinase (HSV1-tk) [17,18]. The *in vivo* labeling used in this study has been previously limited to myocardial metabolic functioning, tumor proliferation, and select inflammatory processes.

The study of the post-pneumonectomy lung also highlights a limitation of semi-quantitative FDG activity analyses such as SUV. In most circumstances, SUV provides a normalized measure of the average FDG activity per unit volume [19]. After pneumonectomy, however, lung volume increases and tissue density, at least initially, decreases. The result is that SUV likely underestimated the FDG activity in the right lung. We anticipate that with increased resolution of pre-clinical PET scanners, it will be possible to precisely measure activity per three-dimensional volume of lung (voxels), that is, regions of interest will accurately identify entire lobes. We speculate that these studies will demonstrate statistically significant glucose uptake in all four lobes of the right lung.

The theoretical applications of FDG-PET include any tissue with a high metabolic consumption of glucose. FDG-PET uses a radiolabeled glucose analogue with the positron-emitting radioactive isotope fluorine-18 substituted for a normal hydroxyl group in the glucose molecule. A substrate for hexokinase-associated phosphorylation, the phosphorylated FDG molecule is trapped within the cell and provides a mechanism for relatively stable tracer accumulation [15]. Notably, an experimental advantage of studying lung regeneration is that the low baseline consumption of glucose provided a useful contrast between control and regenerating lungs.

The peak of detectable glucose metabolism on day 14 after murine pneumonectomy is consistent with known lung cell dynamics. Flow cytometry has demonstrated peak proliferation of endothelial cells [10], alveolar macrophages [20], and whole lung digests [11] 6 to 7 days after pneumonectomy. Likely reflecting progressive cellular accumulation, the total cell numbers of epithelial cells [21], alveolar macrophages [20], and migratory CD11b cells continue to increase until 14 days after pneumonectomy. We suspect that these cells, actively participating in lung remodeling, have significantly elevated glucose metabolism and contribute to the FDG signal detected in the cardiac lobe.

The technical requirements for murine FDG-PET included sufficient anesthesia to produce a motionless mouse throughout the imaging procedure. In contrast to humans, sedation in mice results in rapid oxygen desaturation. The sedation-induced hypoxemia in mice is a consequence of their hypercompliant chest wall and active metabolism [12]. Awake mice use breathing frequency and tidal volume to maintain functional residual capacity and blood oxygenation. When mice are sedated, their respiratory rate is lowered, resulting in lung volume loss and significant hypoxemia. Whereas the hypoxemia can be ameliorated with supplemental oxygen, the loss of lung volume becomes a major obstacle to accurate pulmonary imaging.

The low lung volumes associated with sedation (without ventilation) in mouse studies is also associated with an underestimation of FDG uptake within the lung. Since regions such as the cardiac lobe are only about 5 mm in maximum diameter, the extremes of the lobe (tips) approach the spatial resolution of the PET scanner. The practical consequence is that the measured activity concentration in these regions is significantly less than the true activity concentration. In addition, the small regions of interest are associated with increased image noise and larger random errors in measured tracer uptake. To address these problems, we used orotracheal intubation, inhalational anesthesia (isoflurane), and pressure-controlled ventilation to obtain our PET/CT scans. Controlled inspiratory pressures of 20 cmH_2_O (mid-thoracic volume) led to reproducible scans across experimental groups. We routinely use pressure-controlled ventilation in mouse studies, either PET or CT, requiring anatomic definition of the lung.

## Conclusions

We conclude that the cardiac lobe is the dominant contributor to compensatory growth after murine pneumonectomy. Further, PET/CT scanning can detect both the volumetric increase and the metabolic changes associated with the regenerative growth in the murine cardiac lobe.

## Abbreviations

3D: Three-Dimensional; Bq: Becquerel; CT: Computerized Tomography; FDG: 2-[fluorine-18]fluoro-2-deoxy-d-glucose; LWI: Lung Weight Index; MBq: Megabecquerel; PET: Positron-Emission Tomography.

## Competing interests

The authors declare that they have no competing interest.

## Authors’ contributions

BCG participated in the study design, performed the experiments and data analysis, and edited the manuscript. MP participated in the study design and coordination and reviewed the data analysis. KC participated in the experiments and data analysis. AY participated in the data analysis. MAK participated in study design and manuscript editing. AT participated in the data analysis and manuscript editing. SJM conceived of the study, participated in the design and coordination, reviewed the data analysis, and edited and approved the final manuscript. All authors read and approved the final manuscript.
